# Micronutrient Deficiencies, Nutritional Status and the Determinants of Anemia in Children 0–59 Months of Age and Non-Pregnant Women of Reproductive Age in The Gambia

**DOI:** 10.3390/nu11102275

**Published:** 2019-09-23

**Authors:** Nicolai Petry, Bakary Jallow, Yankuba Sawo, Momodou K. Darboe, Samba Barrow, Aminatta Sarr, Pa Ousman Ceesay, Malang N. Fofana, Andrew M. Prentice, Rita Wegmüller, Fabian Rohner, Modou Cheyassin Phall, James P Wirth

**Affiliations:** 1 GroundWork, 7306 Fläsch, Switzerland; rita@groundworkhealth.org (R.W.); fabian@groundworkhealth.org (F.R.); james@groundworkhealth.org (J.P.W.); 2 National Nutrition Agency, Banjul, The Gambia; bakaryjallow24@yahoo.co.uk (B.J.); kekendoo@yahoo.com (M.N.F.); modoucheyassinphall@yahoo.com (M.C.P.); 3 UNICEF, Banjul, The Gambia; ysawo@unicef.org (Y.S.); asarr@unicef.org (A.S.); 4 Medical Research Council Unit The Gambia at London School of Hygiene and Tropical Medicine, Atlantic Boulevard, Fajara, Banjul, The Gambia; mdarboe@mrc.gm (M.K.D.); andrew.prentice@lshtm.ac.uk (A.M.P.); 5 Gambia Bureau of Statistics, Banjul, The Gambia; barrowmannasy@yahoo.com (S.B.); dadyousman@gmail.com (P.O.C.)

**Keywords:** The Gambia, national cross-sectional survey, micronutrient deficiency, anemia determinants, iron deficiency, vitamin A deficiency, undernutrition, stunting, head circumference, wasting

## Abstract

Data on micronutrient deficiency prevalence, nutrition status, and risk factors of anemia in The Gambia is scanty. To fill this data gap, a nationally representative cross-sectional survey was conducted on 1354 children (0–59 months), 1703 non-pregnant women (NPW; 15–49 years), and 158 pregnant women (PW). The survey assessed the prevalence of under and overnutrition, anemia, iron deficiency (ID), iron deficiency anemia (IDA), vitamin A deficiency (VAD), and urinary iodine concentration (UIC). Multivariate analysis was used to assess risk factors of anemia. Among children, prevalence of anemia, ID, IDA, and VAD was 50.4%, 59.0%, 38.2%, and 18.3%, respectively. Nearly 40% of anemia was attributable to ID. Prevalence of stunting, underweight, wasting, and small head circumference was 15.7%, 10.6%, 5.8%, and 7.4%, respectively. Among NPW, prevalence of anemia, ID, IDA and VAD was 50.9%, 41.4%, 28.0% and 1.8%, respectively. Anemia was significantly associated with ID and vitamin A insufficiency. Median UIC in NPW and PW was 143.1 µg/L and 113.5 ug/L, respectively. Overall, 18.3% of NPW were overweight, 11.1% obese, and 15.4% underweight. Anemia is mainly caused by ID and poses a severe public health problem. To tackle both anemia and ID, programs such as fortification or supplementation should be intensified.

## 1. Introduction

Anemia and malnutrition affect large parts of the world’s population with the highest occurrences in countries located in Sub-Saharan Africa [1]. Young children and women of reproductive age are the most vulnerable population groups to vitamin A, iodine, and iron deficiencies and anemia [2,3]. Globally anemia can be found in more than one-third of women and more than 40% of children under five years of age [1]; primarily in those living in rural households with low socioeconomic status and exposed to poor sanitation [4,5]. Mild forms of micronutrient malnutrition and anemia can have consequences for health and development and severe anemia is often associated with increased maternal mortality, pre-mature birth, low birth weight, and impaired child development [2,3]. Anemia is considered a severe public health problem if the prevalence in a population is ≥40% [6]. As of 2011, anemia posed a severe public health problem among children under five years of age in 69 of the 181 WHO member states and among women in 32 WHO member states [7], causing 8.8% of the global total years lived with disability, primarily in Sub-Saharan Africa [8]. Reasons for anemia are manifold and differ by region and country depending on geographic location and thus climate, household wealth, nutrition, and sanitary conditions [2,8,9]. The most common causes of anemia are micronutrient deficiencies, particularly iron, vitamin A, folate, and vitamin B12 deficiencies [2,4,10]. In addition, malaria and genetic hemoglobin disorders are main contributors to the anemia burden [2]. Due to the wide range of factors causing anemia it is not possible to implement targeted interventions without assessing the determinants of anemia in the region of interest beforehand. While The Gambia has some data on the nutritional and micronutrient status of women and young children, national-level data is outdated and there is no comprehensive survey assessing both micro- and macro-nutrient status. In 2013, about 73% of children and 58% of non-pregnant women of reproductive age were anemic [11], but key risk factors of anemia, such as iron deficiency, malaria, and inflammation have not been measured. The 2018 Gambian Micronutrient Survey (GMNS) was conducted among children and women to assess iron, vitamin A, and iodine status together with undernutrition and anemia and its underlying risk factors in order to formulate new strategies to combat anemia and malnutrition in The Gambia. This paper reports findings on the nutritional status, micronutrient deficiency prevalence, and determinants of anemia in children and women from the 2018 GMNS survey [12].

## 2. Materials and Methods

### 2.1. Survey Design and Participants

The GMNS was a nationwide cross-sectional stratified survey. Proportions were calculated to derive the prevalence of key nutrition and micronutrient indicators in children 0–59 months of age and non-pregnant women. All measures of precision accounted for the complex cluster design and stratified sampling. Two explicit strata were established: 1) urban and 2) rural areas in The Gambia. In addition, implicit sampling was done by urban and rural areas in each Local Government Area (LGA), so that 14 strata from across eight LGAs were established. The 2018 Multiple Indicator Cluster Survey (MICS) served as a sampling frame for the GMNS (the Banjul and Kanifing LGAs only contained urban areas). A two-stage sampling procedure was conducted to select households at random. First, enumeration areas (EAs) within each of the 14 strata were randomly selected with probability proportional to population size from the 390 EAs included in the MICS. Second, the GMNS randomly selected a subsample of the 20 households enrolled by the MICS in each EA. All children (0–59 months of age), non-pregnant women (15–49 years of age), and pregnant women living in the selected households were included in the survey. Anthropometry was taken from all individuals. Blood was taken from a sub-sample of children and non-pregnant women, and from all pregnant women.

### 2.2. Data Collection

Prior to data collection, experienced field workers received intensive classroom training, laboratory practice, and field testing of all survey procedures. Two anthropometrists per team were trained on anthropometric measurements, and an anthropometry standardization exercise was conducted. Phlebotomists were trained on blood and urine collection techniques as well as blood collection procedures such as the correct labeling of samples, and maintenance of the cold chain when collecting and transporting blood specimens. 

A short household questionnaire was administered first to the head of the household or another knowledgeable adult household member. The household questionnaire contained modules related to household food purchase and consumption. Short questionnaires were administered to all recruited women, collecting information on consumption of and knowledge about fortified and fortifiable foods as well as on physical activity. For children, no questionnaire was administered. 

### 2.3. Anthropometric Measurements 

**Figure 1:**
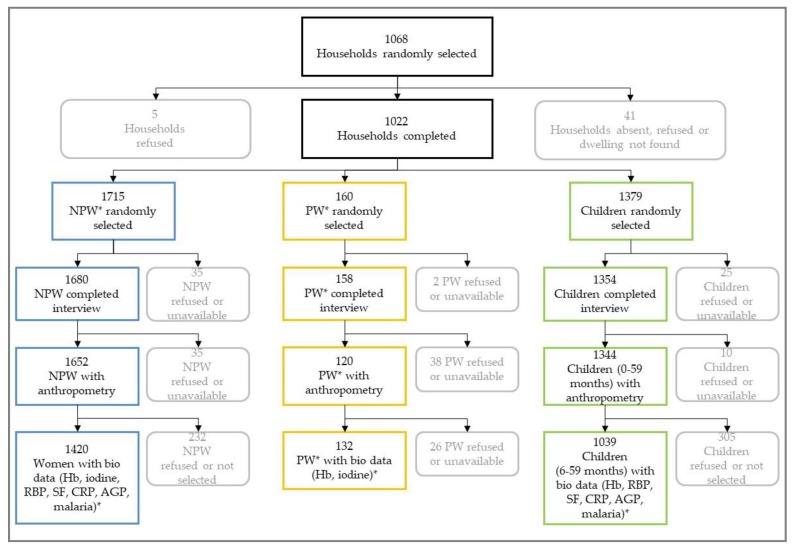
Participation flowchart of households, women and children, Gambian Micronutrient Survey (GMNS 2018). * AGP, alpha-1 acid glycoprotein; CRP, c-reactive protein; Hb, hemoglobin; NPW, non-pregnant women; PW, pregnant women; RBP, retinol binding protein; SF, serum ferrtitin.

Anthropometric measurements were done on all participants (Figure 1). Weight was measured using a medical scale (SECA, Hamburg, Germany). If a child was not able to stand on the scale alone, weight was first taken from the mother or caregiver. Following, the combined mother-and-child weight was taken, so that the child’s weight was obtained by subtracting the mother’s weight from the combined weight using the tare function of the scale. Children and women were weighed with light clothing. For children and women, length/height measurements were taken using a standard wooden height/length board (UNICEF, Copenhagen, Denmark); for those children younger than two years old, recumbent length was measured. Head circumference in children was measured by using a circumference measuring tape (SECA 201CM, Hamburg, Germany). For pregnant women, only their MUAC was measured by using MUAC tapes (UniMUAC tapes, Médecins sans Frontières, UK).

### 2.4. Biofluid Collection and Laboratory Analyses

Capillary blood was collected from all children 6–59 months, non-pregnant, and pregnant women; no blood was taken from children younger than six months of age (Figure 1). Heel-prick was used for children 6–11 months of age and finger prick for children 12–59 months of age to collect the blood sample. The finger or heel was cleaned with alcohol and wiped dry with a sterile gauze pad. Following the lancet puncture (Becton Dickinson, Franklin Lakes, NJ, USA), the first drop was wiped away. The second drop of blood was used to measure hemoglobin concentration using a portable hemoglobinometer (Hb301+, HemoCue™, Angelsholm, Sweden). HemoCue quality control was conducted every morning using three different levels (low, normal, and high) of control blood (Eurotrol, Ede, Netherlands).The third drop was collected to measure malaria status using a rapid diagnostic test kit (RDT) (SD Bioline, Malaria Ag P.f/Pan, Standard Diagnostics Inc, Gyeonggi-do, Republic of Korea). Following, 300–400 µl of blood was collected from children and non-pregnant women into a silica-coated blood collection tube (Sarstedt, Microvette^®^ 300 Z). About 50 ml of urine was collected from all non-pregnant and pregnant women into urine beakers. 

Following on-site measurements, the labeled microtainers and urine beakers were placed in a cool box at 2–8 °C in the dark until transport for processing in the evening of the same day in one of the four regional laboratories in Fajara, Keneba, Basse, and Farafenni. In the regional laboratories, samples were centrifuged at 3,000 rpm for seven minutes to separate the serum, which was then aliquoted into appropriately labeled cryovials. Aliquots were stored at −80 °C except in the Farafenni Lab where they were stored at −20 °C until at the end of the fieldwork when they were transported to Fajara for storage at −80 °C before they were shipped on dry ice to an international laboratory.

Serum for children and women was analyzed for retinol-binding protein (RBP), ferritin, C-reactive protein (CRP), and a1-acid glycoprotein (AGP) at the VitMin-Lab (Wilstaett, Germany) using an ELISA method [13]. Sub-clinical malaria infection was assessed in the Fajara Lab at the MRC Unit The Gambia @ LSHTM using the red cell pellets from children and non-pregnant women. Sub-clinical malaria was assessed by extracting DNA [14] and determining presence of *Plasmodium falciparum* using the ultra-sensitive quantitative polymerase chain reaction (PCR) methods [15]. Urinary iodine concentration was determined at the Iodine Global Network Laboratory at the Noguchi Memorial Institute for Medical Research in Accra, Ghana using the ammonium persulfate/Sandell-Kolthoff reaction method [16].

### 2.5. Parameters and Clinical Thresholds

Hemoglobin concentrations were adjusted for smoking status according to World Health Organization (WHO) recommendations [17]; no adjustment of altitude was required as the highest altitude in The Gambia is 53 meters. Anemia in pregnant women and children was defined as hemoglobin concentration <110 g/L. Severe, moderate, and mild anemia was defined as hemoglobin concentration <70, 70–99, and 100–109 g/L, respectively. Non-pregnant women were classified anemic if the hemoglobin concentration was <120 g/L. Non-pregnant women with concentrations of <80, 80–109, and 110–119 g/L were classified with severe, moderate, and mild anemia, respectively [17]. National weighted anemia prevalence in children and women were used to assess the severity of the public health problem posed by anemia according to WHO criteria [17].

Ferritin concentrations were adjusted for inflammation using CRP and AGP levels using the method developed by the BRINDA project [18]. Ferritin concentrations <12 μg/L and <15 μg/L defined iron deficiency (ID) in children and women, respectively [19]. For children, RBP concentrations were also adjusted for CRP and AGP using the BRINDA adjustment, and the RBP concentrations in women were not adjusted for inflammation as suggested by the BRINDA project [20]. In children, RBP concentrations <0.7 µM/L were used to define vitamin A deficiency. Because RBP is not a WHO-recommended biomarker for vitamin A status assessment, retinol was measured in a sub-sample of children by high-performance liquid chromatography (HPLC) at the VitMin-Lab. There was very good agreement between retinol and RBP values upon linear regression, which confirmed the use of 0.7 µM/L cutoff in children. As only few women were found to be vitamin A deficient, we used a cut-off of 1.05 μmol/L, which has been suggested to define vitamin A insufficiency in women [21].

Thresholds for elevated CRP and AGP were >5 mg/L and >1 g/L, respectively. The inflammatory stage was grouped as follows: No inflammation, elevated CRP only, elevated CRP and AGP, and elevated AGP only [22]. 

Different cut-offs were used to define iodine deficiency depending on whether women were pregnant or not [23]. Individual women were not classified as deficient or sufficient since an individual’s spot urinary iodine concentration shows large diurnal variation and thus, cannot accurately measure deficiency [23]. 

Child undernutrition (including wasting, stunting, underweight, and microcephaly) and overnutrition was defined using WHO Child Growth Standards: z-scores of ≤−2.0 for weight-for-height, height-for-age, weight-for-age, and head circumference-for-age were defined as wasted, stunted, underweight, or having microcephaly, respectively [24,25]. Irrespective of their weight-for-height z-score, children with bilateral pitting edema in the feet and/or lower legs were automatically considered as having severe acute malnutrition. Overnutrition was defined as a weight-for-height z-score greater than +2.0. Overweight was defined as a weight-for-height z-score of greater than +2.0 but less than or equal to +3.0 and obesity as a weight-for-height z-score greater than +3.0.

In non-pregnant women, chronic energy deficiency and overnutrition was assessed using body mass index (BMI; kg/m^2^). BMI thresholds were as follows: <16.0 severe chronic energy deficiency; 16.0–16.9 moderate chronic energy deficiency; 17.0–18.4 at-risk for energy deficiency; 18.5–24.9 normal; 25.0–29.9 overweight; and ≥30.0 obese [26].

### 2.6. Data Management and Statistical Analysis

Data were collected using tablet computers and was entered directly into CSPro. Data analysis was done using Stata/IC version 14.2 (Stata Corp., College Station, TX, USA). Statistical weights were applied to all data to account for the unequal probability of selection in the 14 sub-strata. For continuous variables, means and medians were calculated with corresponding confidence intervals. For urinary iodine concentration (UIC), weighted medians were calculated using Stata’s *epctile* command [27]. Weighted 95% confidence intervals for UIC were calculated using a two-step process that assumes no effect modification due to the survey’s design. First, unweighted confidence intervals were calculated using a 1000-repetition bootstrap simulation. Second, the unweighted confidence intervals were subtracted from the weighted median to yield weighted confidence intervals [28]. *p*-values to compare UICs in rural and urban areas were calculated using linear regression with the square-root of UIC as the dependent variable and residence the independent variable. For categorical variables, proportions were calculated to derive the prevalence of the different outcomes. The statistical precisions of prevalence estimations were assessed by using 95% confidence limits. Measures of precision, including confidence intervals and chi-square *p*-values for differences, took into account the complex cluster and stratified sampling used by the survey. 

Descriptive statistics were calculated for children and women across all strata (i.e., national estimate), for each stratum separately. Factors associated with anemia in children and women were identified using bivariate analyses. All variables statistically significantly associated with anemia in bivariate analyses were included into the multi-variate model after checking for co-linearity. We included the remaining covariates into a block stepwise Poisson regression model and removed non-significant blocks to achieve concise final regression models [29]. The Poison regression produced adjusted risk ratios (aRRs), which were compared with crude and unweighted risk ratios. The population attributable fraction (PAF) for each factor remaining in the model was calculated by using the aRRs produced during the final regression model and the proportion of anemia cases with exposure to the risk factor [30].

### 2.7. Ethics and Consent

Ethical approval for the survey (R18014) was obtained from The Gambia Government / MRC Joint Ethics Committee and the School of Medicine and Allied Health Sciences Research & Publication Committee (The Republic Committee), University of The Gambia. Oral consent was obtained for household interviews from the household head or another knowledgeable person. Written informed consent was obtained from all participating women who were at least 18 years of age. For all participating children and women younger than 18 years, written informed consent was sought from the parent/caregiver. For consenting but illiterate participants, the consent form was read out loud to them and a fingerprint was taken as evidence of consent in lieu of a signature or the participants assigned a witness to sign on their behalf. Survey respondents diagnosed with severe anemia, severe acute malnutrition, and/or malaria (determined using the rapid test kit) were referred to the local health facility for further diagnosis and treatment.

## 3. Results

### 3.1. Children

Of the 1354 children 0–59 months enrolled in the GMNS, 51.1% were male and 48.9% female. From the age groups 0–5 months and 6–11 months 143 and 124 children were recruited, representing 10.6% and 9.2% of the survey sample. For the other age groups the distribution was as follows: 273 children aged 12–23 months (20.2%), 266 children aged 24–35 months (19.6%), 297 children aged 36–47 months (21.9%), and 251 children aged 48–59 months (18.5%). While more children from rural areas were enrolled (rural: 804 versus urban: 550), rural children represented about 37% of the total sampling following statistical weighting, due to the large proportion of the Gambian population residing in urban areas.

#### 3.1.1. Anthropometric and Micronutrient Indicators

The national prevalence and prevalence by urban/rural strata for anthropometric and micronutrient indicators are presented in Table 1. While the national prevalence of stunting, wasting, and underweight were relatively low, the prevalence of stunting, wasting, underweight, and microcephaly was found to be about double in rural areas compared to urban centers. About half of the children 6–59 months of age in The Gambia were anemic, most of them suffering from mild (28%, 95% CI: 24.2, 32.3) or moderate (21.4%; 95% CI: 18.0, 25.4) anemia; very few children had severe anemia. The prevalence of anemia, ID, and iron deficiency anemia (IDA) were higher in rural areas; more than two-thirds of the children in rural households had ID and every fourth child had IDA. Similar differences between urban and rural were also found for vitamin A deficiency prevalence. Nearly 15% of urban children were vitamin A deficient compared to 25% in rural children (Table 1). Though malaria was only observed in one child using RDT, 8.0% of surveyed children were found to have sub-clinical malaria infection using PCR analysis. 

#### 3.1.2. Bivariate Analyses of Anemia Risk Factors

Age was identified as one of the risk factors for anemia (*p* < 0.005). In children aged 6–35 months anemia prevalence was about twice as high compared to children 36–59 months (Table 2). Further, a significantly higher proportion of male children (*p* < 0.05) compared to female children had anemia. Residence (*p* < 0.05) as well as region (*p* < 0.005) were found to be determinants of anemia. Significantly more children residing in rural areas had anemia compared to those living in urban centers and the highest anemia prevalence’s were found in Mansakonko and Kerewan, whereas fewer children living in Banjul and Kanifing had anemia. Household wealth and household sanitation were not associated with anemia prevalence. Of the investigated recent illnesses, only fever was significantly associated with anemia (*p* < 0.05). Anemia prevalence did not significantly differ by wasting status, but children that had either stunting, underweight, or microcephaly had higher levels of anemia. No associations were found between anemia and malaria or diarrhea, or cough in the past two weeks. However, children with elevated CRP and/or AGP had higher levels of anemia compared to children with no inflammation (*p* < 0.05). Consumption of vitamin/mineral supplements and infant formula were significantly associated with anemia; anemia prevalence was about half in children consuming vitamin/mineral supplements and/or infant formula compared to those not consuming. No associations were found between anemia and the consumption of fortified baby foods. ID (*p* < 0.0001) and vitamin A deficiency (VAD) (*p* < 0.005) were highly associated with anemia. More than twice as many children suffering from ID were anemic compared to those without ID, and anemia prevalence in children with VAD was about 12 percentage points higher compared to those sufficient in vitamin A.

#### 3.1.3. Multivariate Analyses

Children with ID had more than twice the risk to develop anemia compared to iron replete children, and nearly 40% of the anemia in children was attributable to ID (Table 3). Other anemia risk factors were vitamin A status, any inflammation, and stunting. Children with VAD had a 21% increased risk for anemia compared to those without VAD, children with inflammation had a 29% increased risk for anemia compared to those without infection and stunted children had a 17% increased risk to develop anemia compared to non-stunted children (Table 3).

### 3.2. Women

In total the survey enrolled 1,875 women of reproductive age. The age distribution was skewed, with a higher proportion of younger women enrolled than older women. Of the included women, 91.6% were non-pregnant and 8.4 pregnant from self-reporting. Almost equal proportions of women were selected from urban and rural areas.

#### 3.2.1. Anthropometric and Micronutrient Indicators

Table 4 presents the prevalence of over and undernutrition indicators, as well as mean BMI and MUAC. The prevalence of undernutrition in non-pregnant women was about 15%, and approximately 8% in pregnant women. The undernutrition prevalence for both groups did not significantly differ by urban or rural residence. Nationally, nearly 30% of non-pregnant women were overweight or obese, with a significantly higher prevalence (*p* < 0.05) found in urban areas. The prevalence of overweight only (18.3%; 95% CI: 15.1, 22.0) was higher than obesity only (11.1%; 95% CI: 9.0, 13.7). There was no significant difference in the prevalence of obesity between urban and rural areas. Anemia was found in more than half of the participating women, with no significant differences by residence. Nationally, about 40% of non-pregnant women had ID and almost 30% had IDA. The prevalence of ID and IDA were significantly higher (*p* < 0.001) in rural areas compared to urban centers. Similarly, significantly more non-pregnant women who lived in rural areas had VAD or vitamin A insufficiency (*p* < 0.001). Median urinary iodine concentration in pregnant women suggested an insufficient iodine status. Iodine status was significantly lower for pregnant women living in rural areas compared to urban areas (Table 4).

#### 3.2.2. Bivariate Analyses of Anemia Risk Factors

Neither women’s age, residence, education, household wealth, or household sanitation were associated with anemia (Table 5). Although not significant, differences in anemia prevalence were large between certain LGAs. The highest prevalence was observed in Kuntaur, where more than two-thirds of the women had anemia; the lowest prevalence was found in Banjul where only one-third of the women were affected. Iron status was highly associated with anemia; almost twice as many women with ID also had anemia, compared to women without ID. Malaria and inflammation were not associated with anemia, but women classified as vitamin A insufficient had significantly higher levels of anemia. Neither the nutritional status (i.e., BMI category) nor adequate dietary diversity were identified as risk factors for anemia. 

#### 3.2.3. Multivariate Analyses

ID was identified as the main risk factor for anemia among women (Table 6). Non-pregnant women with ID had a two-fold higher risk of developing anemia compared to those women without ID; and about 33% of anemia was attributable to ID. The risk of anemia was also 1.2 times higher among vitamin A insufficient women, and 7% of the total anemia observed was attributable to vitamin A insufficiency.

## 4. Discussion

The national prevalence of ID in The Gambia in children and women was found to be 59.0% and 41.4%, respectively. Putting these results into context with other national representative assessments in countries located in Western Sub-Saharan Africa shows that the prevalence in The Gambia is exceptionally high. Surveys conducted in Cameroon [31], Côte d’Ivoire [32], Sierra Leone [33], Liberia [34], and Ghana [35] found ID prevalence’s range between 5–30% in children and between 8–20% in women. All aforementioned surveys adjusted serum ferritin for inflammation according to the approach developed by Thurnham [36], whereas the GMNS adjusted ferritin concentrations using the newly-developed BRINDA method [18]. Despite this methodological difference, the prevalence of ID would nonetheless be higher in The Gambia than in other countries. Using the Thurnham correction method, 54.2% and 36.8% of Gambian children and women would be classified as iron deficient. While our analysis of ID risk factors showed that age and urban/rural residence are associated with ID [12], it is likely that also low iron intake in combination with low iron bioavailability, caused by the high concentration of iron absorption inhibitors present in plant-based diets [37], may be responsible for the poor iron status in The Gambia. In addition, The Gambia has no mandatory iron fortification program, which may further limit the iron consumption of the general population. In order to tackle iron deficiency, programs of mandatory iron fortification of cereals should be implemented along with surveillance strategies to guaranty the enforcements of the programs. Those programs should be tailored to reach the most affected, who are women and children residing in rural areas, such as Kuntaur and Mansakonko as well as women and children living in poor households and women with low education level [12]. Survey findings show that rice and wheat flour constitute suitable iron fortification vehicles as both are consumed by the vast majority of Gambian households [12]. 

The prevalence of vitamin A deficiency in women and children poses no public health problem in women and a moderate public health problem in children according to WHO classification [38]. However, vitamin A deficiency poses a severe public health problem in children living in rural areas as well as in children 24–59 months of age and children living in Janjanbureh, Kuntaur, and Basse and must thus be addressed instantaneously [12]. To fill the nutrient gap, a holistic approach can be considered, including strengthening the coverage of vitamin A supplementation in the near term, and the expansion of vitamin A fortification and promotion of vitamin A-rich foods. Importantly, vitamin A deficiency is only a severe problem in some population groups and any programs aiming to reduce vitamin A deficiency must take into account that vitamin A might be supplied in excess to those groups not deficient and might cause vitamin A toxicity [39]. Thus, programs must be tailored to the needs of The Gambian population targeting the most vulnerable groups.

Iodine deficiency can have serious consequences on growth and development due to inadequate thyroid hormone production [40], pregnant women and young children being the most vulnerable population groups. The median urinary iodine concentration in The Gambia in pregnant women indicates iodine deficiency; particularly among those living in rural areas and in households with inadequately iodized salt [12]. While the national median urinary iodine concentration in non-pregnant women denotes iodine sufficiency, for certain sub-groups of non-pregnant women, such as women residing in Kuntaur and Mansakonko or women living in households of the lowest wealth quintile, the median urinary iodine concentrations indicated deficiency [12]. In 1999, an iodine status assessment was done among Gambian school-age children, finding a median urinary iodine concentration of 42 µg/L, classifying these children as moderately iodine deficient [41]. The results presented here show that past and ongoing efforts did not eradicate iodine deficiency, and that only a small proportion of the salt consumed in The Gambia is adequately iodized [12]. Thus, extending the coverage of iodized salt to all regions and specific target groups and strengthening the salt iodization surveillance system including quality assurance at the point of production and entry could increase iodine intake. 

Nationally, wasting, underweight, and stunting, denote only mild to moderate public health problems in Gambian children [42]. However, stunting, wasting, and underweight are more prevalent in certain sub-groups, such as in children residing in rural areas or in LGAs with a high proportion of rural population [12]. Further, stunting is associated with inadequate household sanitation [12]. This is in accordance with results from other studies, which identified poor sanitary conditions and the sustained exposure to enteric pathogens as risk factor for stunting [43]. However, this survey’s findings demonstrate a decline for all three indicators of malnutrition compared to prior assessments in The Gambia [11]. To our knowledge, this is the first national assessment of microcephaly in Western Africa. Microcephaly, which affects brain size and thus cognitive development [44,45], was found in 7.4% of the surveyed children. Our data shows a strong association between undernutrition and microcephaly in The Gambia, as significantly more stunted, wasted, and underweight children also suffered from microcephaly [12], which is in agreement with previous findings from other countries [44]. 

Undernutrition in women, both pregnant and non-pregnant, is not at worrying levels in The Gambia. Just about 15% of women are undernourished, but of these, two thirds fall in the ‘at risk for chronic energy deficiency’ category, and only a very small proportion have severe chronic energy deficiency. Overweight and obesity are by far the larger problem in this group, affecting about one-third of the surveyed non-pregnant women. High BMIs are especially prevalent in older women, in women living in urban areas and women living in wealthier households [12], which is in agreement with literature [46]. 

About half of the children in The Gambia were found to be anemic. Although this is lower than the reported prevalence in 2013 [11], anemia poses a severe public health problem according to WHO classification [6]. Bivariate analyses showed geographic location, age, iron and vitamin A deficiencies, stunting, and inflammation are associated with anemia in Gambian children. In the multivariate model only iron and vitamin A deficiencies, stunting, and inflammation are identified as risk factors of anemia. About 75% of anemic children had concurrent iron deficiency, which is substantially higher than the 28% estimate in a recent meta-analysis for countries in sub-Saharan Africa [9] and indicates that iron deficiency plays an important role in the etiology of anemia in Gambian children. Although to a lesser extent than iron deficiency, vitamin A deficiency as well as stunting is associated with anemia, clearly suggesting that anemia in Gambian children is mainly nutritionally induced. Anemia of infection, which has been identified to largely contribute to anemia in Sub-Saharan Africa [2] is of minor importance in The Gambia. About 35% of the children had increased inflammation markers at the time of the survey, which is at the lower end of the range compared to other countries in Western Africa [32,33,35,47]. Further, although malaria has been identified as one of the main risk factors of anemia in Sub-Saharan Africa [33], it was almost nonexistent among study participants at the time of the survey. In other countries in West Africa, the malaria prevalence measured using RDT found a prevalence of 5%–50% [32,33,35,47]. However, some of the surveys were conducted shortly after the rainy season where malaria is more prevalent and the GMNS was conducted in the middle of the dry season; thus, malaria infection and consequently anemia prevalence might be higher during or shortly after the rainy season. Overall, inflammation increased the risk for anemia by 30% and 8% of total anemia can be attributed to inflammation. In total, the survey was able to attribute about 50% of the anemia found in children to different risk factors.

The prevalence of anemia found in non-pregnant women and pregnant women was 50.9% and 56.8%, which is, in contrast to children, similar to the prevalence found in 2013 [11]. Multivariate analyses clearly showed that nutritional anemia is the main cause of anemia in women. More than 55% of anemic women had concurrent iron deficiency, and about one-third of anemia can be attributed to iron deficiency. About 8% of anemia was associated with vitamin A insufficiency. Surprisingly, neither inflammation nor malaria was associated with anemia in women, indicating that anemia of infection does not contribute substantially to anemia in that population group. However, a large (60%) proportion of anemia was unexplained by the investigated risk factors. Also, as the GMNS was a cross-sectional survey it was not possible to establish causal mechanisms between any of the outcomes of interest and associated risk factors. Thus, further studies investigating other risk factors such as hemoglobinopathies are warranted to further elucidate the etiology of anemia in Gambian women and children. 

## 5. Conclusions

Anemia and ID are highly prevalent in Gambian women and children, especially in rural areas and certain LGAs. Since anemia is associated primarily with ID and VAD, intensifying Gambia’s fortification or supplementation programs is likely key to addressing anemia and micronutrient deficiencies. For both women and children, a large proportion of anemia remained unexplained by the investigated risk factors. The low malaria prevalence found was likely a result of the dry conditions during data collection. Had the GMNS been implemented in the rainy season, malaria prevalence would likely be higher, and malaria could have been significantly associated with anemia. Future studies that contain a more comprehensive suite of potential anemia determinants and are designed to detect causal relationships are recommended.

## Figures and Tables

**Table 1:** Child anemia micronutrient indicators (6–59 months) and anthropometric measures (0–59 months), The Gambia 2018.

	National			Urban			Rural			
Characteristic ^a^	*N*	% ^b^ / Mean/ Median	95%CI ^c^	*N*	% ^b^ / Mean/ Median	95%CI	*N*	% ^b^ / Mean/ Median	95%CI ^c^	*p*-Value ^d^
Nutritional status ^e^										
Stunting										
HAZ, mean	1337	−0.71	(−0.82, −0.60)	543	−0.57	(−0.69, −0.44)	794	−0.97	(−1.15, −0.78)	<0.001
Any stunting, %	1337	15.7	(13.2, 18.4)	543	11.8	(8.9, 15.3)	794	22.4	(19.1, 26.2)	<0.005
Severe stunting, %	1337	4.9	(3.3, 7.3)	543	3.9	(1.9, −8.0)	794	6.6	(5.1, 8.5)	0.175
Wasting										
WHZ, mean	1338	−0.39	(−0.48, −0.31)	547	−0.28	(−0.37, 0.18)	791	−0.60	(−0.70, −0.49)	<0.0001
Any wasting, %	1338	5.8	(4.1, 8.1)	547	4.4	(2.4, 8.1)	791	8.2	(6.2, 10.7)	0.07
Severe wasting, %	1338	1.2	(0.7, 2.2)	547	0.8	(0.3, 2.3)	791	1.9	(1.0, 3.6)	0.146
Underweight										
WAZ, mean	1344	−0.65	(−0.74, −0.58)	548	−0.51	(−0.59, −0.43)	796	−0.92	(−1.03, −0.81)	<0.0001
Any underweight, %	1344	10.6	(8.4, 13.2)	548	7.8	(5.1, 11.6)	796	15.7	(3.2, 18.6)	<0.005
Severe underweight, %	1344	1.9	(1.2, 3.0)	548	1.0	(0.4, 2.5)	796	3.4	(2.2, 5.2)	<0.05
Head circumference										
Head circumference, mean	1324	−0.47	(−0.55, −0.38)	535	−0.43	(−0.53, −0.33)	789	−0.53	(−0.67, −0.39)	0.234
Microcephaly, %	1324	7.4	(5.1, 10.7)	535	5.8	(2.9, 11.5)	789	10.0	(8.2, 12.1)	0.13
Micronutrient status ^f^										
Hemoglobin concentration										
Hemoglobin (g/L), mean	1014	107.9	(106.7, 109.1)	399	109.3	(107.6, 110.9)	615	105.7	(103.9, 107.5)	<0.001
Any anemia ^f^, %	1014	50.4	(45.9, 54.8)	399	46.0	(40.1, 51.9)	615	57.5	(51.1, 63.6)	<0.05
Severe anemia ^f^, %	1014	0.9	(0.4, 2.1)	399	0.9	(0.2, 3.3)	615	0.9	(0.5, 1.7)	0.9865
Iron status ^g, h^										
Ferritin (µg/L), median	1012	9.17	(7.34, 10.68)	399	10.8	(8.30, 13.23)	613	8.03	(7.40, 8.66)	
Iron deficiency, %	1012	59.0	(51.3, 66.4)	399	54.0	(42.5, 65.1)	613	67.1	(61.2, 72.5)	<0.05
Iron deficiency anemia, %	1007	38.5	(33.7, 43.5)	396	33.8	(27.4, 40.8)	611	46.0	(40.0, 52.0)	<0.01
Vitamin A status ^g, i^										
RBP (µmol/L), mean	1012	0.93	(0.89, 0.97)	399	0.97	(0.91, 1.03)	613	0.87	(0.85, 0.89)	<0.01
Vitamin A deficiency	1012	18.3	(14.5, 22.8)	399	13.8	(8.5, 21.6)	613	25.5	(21.4, 30.0)	<0.05

Note: The n’s are unweighted denominators for each subgroup; subgroups that do not sum to the total have missing data. ^a^ Stunting, wasting, and underweight collected from children 0–59; blood biomarkers and head circumference collected from children 6–59 months of age. ^b^ Percentages weighted for unequal probability of selection. ^c^ CI = confidence interval, calculated taking into account the complex sampling design. ^d^ *p*-value <0.05 indicates that the proportion in urban subgroup is statistically significantly different from the values rural subgroup. ^e^ Severe stunting: height-for-age z-score (HAZ) <−3; any stunting: HAZ <−2; severe wasting: weight-for-height z-score (WHZ) <−3; any wasting: WHZ <−2; severe underweight: weight-for-age z-score (WAZ) <−3; any underweight: WAZ <−2; microcephaly: head-circumference-for-age z-score (HCAZ) <−2. ^f^ Anemia defined in as Hb <110 g/L; severe anemia defined as hemoglobin <80 g/L, respectively. ^g^ Ferritin and RBP concentrations, and associated deficiency prevalence’s, corrected for inflammation according to BRINDA [18,20]. ^h^ Iron deficiency defined as serum ferritin < 12 µg/L; iron deficiency anemia defined as low serum ferritin and low hemoglobin. ^i^ Vitamin A deficiency as RBP <0.7 µmol/L.

**Table 2:** Bivariate associations between anemia in children 6–59 months of age and demographics, nutritional status indicators, and disease.

Characteristic	N	Anemia %^a, b^	(95% CI)^c^	Chi-Square *p*-Value ^d^
Age group (in months)				
6–11	103	64.2	(43.0, 81.0)	<0.005
12–23	230	59.0	(43.7, 72.7)	
24–35	216	63.2	(57.3, 68.7)	
36–47	257	36.2	(28.0, 45.3)	
48–59	208	35.6	(26.4, 46.1)	
Sex				
Male	523	55.4	(48.9, 61.8)	<0.05
Female	491	44.9	(38.8, 51.2)	
Residence				
Urban	399	46.0	(40.1, 51.9)	<0.05
Rural	615	57.5	(51.1, 63.6)	
Region				
Banjul	92	41.9	(32.4, 52.1)	<0.005
Kanifing	90	42.5	(35.8, 49.6)	
Brikama	123	54.7	(44.0, 65.0)	
Mansakonko	148	68.1	(62.2, 73.4)	
Kuntaur	122	57.5	(50.0, 64.7)	
Kerewan	93	60.7	(44.7, 74.6)	
Janjanbureh	182	56.1	(45.3, 66.3)	
Basse	164	50.4	(45.9, 54.8)	
Wealth Quintile				
Lowest	363	58.4	(49.6, 66.8)	0.26
Second	226	51.3	(40.0, 62.6)	
Middle	177	52.5	(37.9, 66.6)	
Fourth	104	45.1	(31.5, 59.5)	
Highest	144	39.0	(29.9, 48.8)	
Household sanitation ^e^				
Inadequate	640	51.9	(45.2, 58.5)	0.41
Adequate	374	48.3	(42.9, 53.7)	
Diarrhea in the past 2 weeks ^f^				
Yes	249	51.7	(42.6, 60.7)	0.79
No	743	50.7	(46.4, 55.0)	
Child had cough in past 2 weeks ^f^				
Yes	225	51.1	(43.8, 58.3)	0.91
No	769	50.5	(44.5, 56.5)	
Child had fever in past 2 weeks ^f^				
Yes	247	57.7	(50.8, 64.4)	<0.05
No	746	48.5	(43.2, 53.7)	
Malaria infection ^g^				
Yes	84	52.1	(43.3,60.9)	0.731
No	912	50.6	(45.9, 55.3)	
Consumed fortified baby foods ^h^				
Yes	44	64.9	(46.8, 79.6)	0.84
No	283	63.0	(50.8, 73.7)	
Consumed vitamin/mineral supplements ^h^				
Yes	39	33.1	(18.0, 52.6)	<0.005
No	327	66.9	(54.4, 77.4)	
Consumed infant formula ^h^				
Yes	14	31.2	(13.5, 56.9)	<0.05
No	312	64.7	(52.6, 75.2)	
Wasting				
Yes (WHZ ≤ −2)	74	51.5	(35.5, 67.2)	0.894
No (WHZ > −2)	933	50.3	(45.7, 55.0)	
Stunting				
Yes (HAZ ≤ −2)	197	65.8	(50.8, 78.2)	<0.05
No (HAZ > −2)	809	47.1	(41.8, 52.5)	
Underweight				
Yes (WAZ ≤ −2)	155	63.1	(50.6, 74.0)	<0.05
No (WAZ > −2)	855	48.6	(44.0, 53.2)	
Microcephaly				
Yes (HCAZ ≤ −2)	92	65.7	(53.6, 76.1)	<0.05
No (HCAZ > −2 to ≤ +2)	918	49.3	(44.5, 54.0)	
Iron status ^i^				
Deficient (serum ferritin < 12 µg/L)	632	65.7	(59.2, 71.6)	<0.0001
Sufficient (serum ferritin ≥ 12 µg/L)	375	29.4	(22.7, 37.1)	
Vitamin A status ^i^				
Deficient (RBP < 0.70 μmol/L)	217	60.6	(51.9, 68.5)	<0.005
Sufficient (RBP ≥ 0.70 μmol/L)	790	48.4	(43.9, 53.1)	
Inflammation ^j^				
None	720	47.7	(43.5, 51.9)	<0.05
CRP and/or AGP elevated	387	57.7	(48.3, 66.7)	

Note: The n’s are unweighted number of children in each subgroup; subgroups that do not sum to the total have missing data. ^a^ Percentages weighted for non-response and survey design. ^b^ Anemia defined as hemoglobin < 110 g/L adjusted for altitude. ^c^ CI = confidence interval, calculated taking into account the complex sampling design. ^d^ *p*-value < 0.05 indicates that the proportion in urban subgroup is statistically significantly different from the values rural subgroup. ^e^ Composite variable of toilet type and if toilet facilities are shared with non-household members; Adequate sanitation = flush or pour flush toilet or pit latrine with slab not shared with another household. Inadequate sanitation = open pit, bucket latrine, hanging toilet/latrine, no facility, bush, field ^f^ Two weeks prior to the MICS survey. ^g^ Malaria detected via detection of the cytochrome oxidase III (COX-III) gene of Plasmodium parasites. ^h^ Dietary consumption at time of the MICS survey; indicators only collected in children 6–23 months of age. ^i^ Ferritin and RBP concentrations, and associated deficiency prevalence’s, corrected for inflammation according to BRINDA [18,20]. ^j^ Elevated CRP and AGP defined as >5 mg/L and>1 g/L, respectively.

**Table 3:** Crude and adjusted relative risk of anemia in children (6–59 months of age), The Gambia 2018.

Characteristic	Category	*N*	Crude (Bivariate Analysis)		Adjusted (Poisson Regression) ^a^		Population Attributable Fraction ^b^
			Relative Risk	95% CI	Relative Risk	95% CI	
					*N* = 1012		
Iron status	Deficient	645	1.82	(1.63, 2.03)	2.11	(1.79, 2.49)	39.4%
	Not deficient	386	referent		referent		
Vitamin A status	Deficient	220	1.51	(1.18, 1.92)	1.21	(1.08, 1.37)	3.8%
	Not deficient	811	referent		referent		
Inflammation	Yes	292	1.67	(1.35, 2.05)	1.29	(1.17, 1.44)	7.6%
	No	739	referent		referent		
Stunting	Yes	198	1.68	(1.29, 2.19)	1.17	(1.00, 1.26)	2.4%
	No	821	referent		referent		

^a^ Child’s age in months as a continuous covariate was included in the child regression model. ^b^ Calculated using relative risk from Poisson regression and the proportion of anemia among exposed (e.g., iron deficient) individuals.

**Table 4:** Micronutrient and anthropometric indicators of women, The Gambia 2018.

	National			Urban			Rural			
Characteristic	N	% ^a^/ Mean/ Median	95%CI ^b^	N	% ^a^ / Mean/ Median	95%CI ^b^, IQR	N	% ^a^ / Mean/ Median	95%CI ^b^	*p*-Value ^c^
Non- pregnant women										
Nutritional status ^d^										
BMI, mean	1651	23.2	(22.8, 23.8)	872	23.5	(23.0, 24.1)	779	22.6	(21.7, 23.6)	0.103
Underweight										
Any underweight, %	1651	15.4	(12.4, 19.0)	872	14.6	(10.8, 19.4)	779	17.3	(13.3, 22.2)	0.375
Severe underweight, %	1651	1.4	(0.8, 2.6)	872	1.4	(0.6, 3.1)	779	1.5	(0.8, 3.1)	0.907
Overweight or obesity										
Overweight and obesity, %	1651	29.7	(25.7, 34.0)	872	32.6	(28.0, 37.7)	779	22.8	(16.0, 31.3)	<0.05
Micronutrient status										
Hemoglobin concentration										
Hemoglobin (g/L), mean	1420	118.5	(116.9, 120.0)	787	119.1	(117.1, 121.1)	643	116.8	(114.6, 119.0)	0.126
Any anemia ^f^, %	1420	50.9	(45.5, 56.4)	787	49.9	(42.8, 57.1)	643	53.5	(47.2, 59.7)	0.45
Severe anemia ^f^, %	1420	1.3	(0.7, 2.6)	787	1.0	(0.3, 3.2)	643	2.0	(1.0, 4.3)	0.309
Iron status ^g, h^										
Ferritin (µg/L), median	1401	18.6	(17.4, 19.8)	764	21.5	(19.6, 23.4)	637	13.9	(11.5, 16.2)	-
Iron deficiency, %	1401	41.4	(37.6, 45.4)	764	37.0	(32.3, 41.9)	637	52.6	(45.2, 60.0)	<0.001
Iron deficiency anemia, %	1392	28.1	(24.5, 32.1)	756	24.5	(20.4, 29.2)	636	37.3	(30.7, 44.3)	<0.01
Vitamin A status ^i^										
RBP (µmol/L), mean	1391	1.36	(1.31, 1.41)	764	1.42	(1.35, 1.48)	637	1.22	(1.14, 1.29)	<0.001
Vitamin A deficiency	1391	1.8	(1.2, 2.8)	764	0.6	(0.3, 1.4)	637	4.9	(2.9, 8.0)	<0.0001
Vitamin A insufficiency	1391	21.6	(18.2, 25.4)	764	17.6	(14.0, 21.9)	637	31.7	(25.5, 38.8)	<0.001
Iodine status										
Urinary iodine (µg/L), median	1287	156.2	(149.4, 163.0)	716	163.6	(154.6, 172.6)	571	126.6	(114.3, 138.9)	0.283
Pregnant women										
Nutritional status										
Mean MUAC	120	26.6	(25.8, 27.4)	50	27.6	(26.1, 29.1)	70	26.0	(25.2, 26.8)	0.28
Undernourished, MUAC <23 cm, %	120	7.5	(4.1, 13.4)	50	6.0	(2.0, 16.8)	70	8.6	(4.1, 16.9)	0.58
Micronutrient status										
Hemoglobin concentration										
Hemoglobin (g/L), mean	132	107.3	(104.4, 110.1)	62	110.0	(105.4, 114.7)	70	104.8	(101.6, 107.9)	0.065
Any anemia ^j^, %	132	56.8	(47.2, 66.0)	62	50.0	(35.5, 64.5)	70	62.9	(50.9, 73.4)	0.18
Iodine status										
Urinary iodine (µg/L), median	118	113.5	(50.1, 205.9)	54	136.5	(92.6, 236.7)	64	88.8	(41.3, 162.8)	<0.05

Note: The n’s are unweighted denominators for each subgroup; subgroups that do not sum to the total have missing data. ^a^ Percentages weighted for unequal probability of selection. ^b^ CI = confidence interval, calculated taking into account the complex sampling design. ^c^ *p*-value < 0.05 indicates that the proportion in urban subgroup is statistically significantly different from the values rural subgroup. ^d^ Severe underweight: BMI <16.0; any underweight: BMI <18.5. ^f^ Anemia defined in as Hb <120 g/L; severe anemia defined as hemoglobin <80 g/L, respectively; after adjusting hemoglobin for smoking. ^g^ Ferritin concentrations, and associated deficiency prevalence’s, were corrected for inflammation according to BRINDA [18]. ^h^ Iron deficiency defined as serum ferritin <15 µg/L; iron deficiency anemia defined as low serum ferritin and low hemoglobin. ^i^ Vitamin A deficiency as RBP <0.7 µmol/L. RBP was not adjusted for inflammation. ^j^ Anemia defined in as Hb <110 g/L; after adjusting hemoglobin for smoking.

**Table 5:** Bivariate associations between anemia in non-pregnant women and demographics, nutritional status indicators and disease, The Gambia 2018.

Characteristic	Number of Women	Anemia %^a, b^	(95% CI)^c^	Chi-Square *p*-Value ^d^
Age group (in years)				
15–19	337	41.0	(34.6, 47.7)	0.06
20–24	263	51.5	(42.9, 60.1)	
25–29	231	55.3	(44.4, 65.7)	
30–34	198	58.3	(45.6, 70.0)	
35–39	188	46.8	(39.6, 54.2)	
40–44	116	62.4	(45.5, 76.7)	
45–49	87	49.1	(36.6, 61.7)	
Residence				
Urban	787	49.9	(42.8, 57.1)	0.45
Rural	643	53.5	(47.2, 59.7)	
Region				
Banjul	224	32.3	(24.2, 41.7)	0.05
Kanifing	202	44.1	(37.6, 50.7)	
Brikama	185	53.6	(42.1, 64.8)	
Mansakonko	164	59.3	(54.3, 64.1)	
Kuntaur	113	67.0	(60.0, 73.3)	
Kerewan	148	37.9	(25.2, 52.6)	
Janjanbureh	201	50.8	(40.0, 61.5)	
Basse	193	57.1	(47.0, 66.6)	
Woman’s Education				
Never at school	590	58.2	(53.6, 62.8)	0.06
Primary school	220	46.1	(35.1, 57.4)	
Lower Secondary	269	47.2	(36.6, 58.1)	
Upper Secondary	227	44.5	(33.4, 56.2)	
Higher	93	48.2	(36.5, 60.2)	
Wealth Quintile				
Lowest	350	60.1	(51.5, 68.1)	0.36
Second	266	52.7	(43.2, 62.1)	
Middle	249	50.3	(44.2, 56.3)	
Fourth	196	46.7	(28.9, 65.4)	
Highest	369	46.5	(39.6, 53.7)	
Household sanitation ^e^				
Inadequate	794	52.0	(46.2, 57.8)	0.51
Adequate	636	49.7	(42.6, 56.8)	
Iron status ^f^				
Deficient (serum ferritin < 12 µg/L)	628	67.8	(60.8, 74.1)	<0.0001
Sufficient (serum ferritin ≥ 12 µg/L)	764	37.4	(30.1, 45.4)	
Vitamin A status				
Insufficient (RBP < 1.05 μmol/L)	370	64.5	(56.4, 71.9)	<0.01
Sufficient (RBP ≥ 1.05 μmol/L)	1022	46.0	(39.1, 53.3)	
Malaria infection ^g^				
Yes	72	57.9	(42.4, 72.0)	0.328
No	1184	50.5	(43.9, 57.0)	
Inflammation ^h^				
None	1143	50.1	(43.4, 56.7)	0.99
CRP and/or AGP elevated	249	49.9	(42.0, 57.9)	
Nutritional status				
Underweight (BMI < 18.5)	224	53.1	(41.3, 64.6)	0.70
Normal weight (BMI 18.5–24.9)	781	51.5	(45.5, 57.4)	
Overweight/Obesity (BMI > 25)	391	49.2	(41.3, 57.2)	
Minimal dietary diversity				
Yes (≥ 5 food groups)	991	49.8	(44.9, 54.8)	0.53
No (0–4 food groups)	415	52.7	(42.8, 62.4)	

Note: The n’s are unweighted number of children in each subgroup; subgroups that do not sum to the total have missing data. ^a^ Percentages weighted for non-response and survey design. ^b^ Anemia defined as hemoglobin <120 g/L adjusted for smoking. ^c^ CI = confidence interval, calculated taking into account the complex sampling design. ^d^ *p*-value < 0.05 indicates that the proportion in urban subgroup is statistically significantly different from the rural subgroup. ^e^ Composite variable of toilet type and if toilet facilities are shared with non-household members; Adequate sanitation = flush or pour flush toilet or pit latrine with slab not shared with another household. Inadequate sanitation = open pit, bucket latrine, hanging toilet/latrine, no facility, bush, field ^f^ Ferritin concentrations and associated deficiency prevalence’s, were corrected for inflammation according to BRINDA [18]. ^g^ Malaria detected via detection of the cytochrome oxidase III gene of Plasmodium parasites. ^h^ Elevated CRP defined as >5 mg/L and elevated AGP defined as >1 g/L.

**Table 6:** Crude and adjusted relative risk of anemia in non-pregnant women, The Gambia 2018.

Characteristic	Category	*N*	Crude (Bivariate Analysis)		Adjusted (Poisson Regression)		Population Attributable Fraction ^a^
			Relative Risk	95% CI	Relative Risk	95% CI	
					*N* = 1422		
Iron status	Deficient	641	2.37	(2.08, 2.71)	2.08	(1.85, 2.34)	33.4%
	Not deficient	781	referent	-	referent		
Vitamin A status	Insufficient	380	1.92	(1.59, 2.28)	1.26	(1.14, 1.39)	7.3%
	Not insufficient	1042	referent	-	referent		

^a^ Calculated using relative risk from Poisson regression and the proportion of anemia among exposed (e.g., iron deficient) individuals.
